# Distinguishing glioblastoma progression from treatment-related changes using DTI directionality growth analysis

**DOI:** 10.1007/s00234-024-03450-8

**Published:** 2024-08-17

**Authors:** R. van den Elshout, B. Ariëns, M. Esmaeili, B. Akkurt, M. Mannil, F. J. A. Meijer, A. G. van der Kolk, T. W. J. Scheenen, D. Henssen

**Affiliations:** 1 Department of Medical Imaging, Radboud University Medical Center, Geert Grooteplein Zuid 10, Nijmegen, 6525 GA the Netherlands; 2 AmsterdamUMC, Radiology and Nuclear Medicine, Amsterdam, Netherlands; 3 Department of Diagnostic Imaging, Akershus University Hospital, Lørenskog, Norway; 4 Department of Electrical Engineering and Computer Science, University of Stavanger, Stavanger, Norway; 5 University Clinic for Radiology, Westfälische Wilhelms-University Muenster and University Hospital Muenster, Muenster, Germany

**Keywords:** Glioblastoma, MRI, DTI, Lesion development, Satellitosis

## Abstract

**Background:**

It is difficult to distinguish between tumor progression (TP) and treatment-related abnormalities (TRA) in treated glioblastoma patients via conventional MRI, but this distinction is crucial for treatment decision making. Glioblastoma is known to exhibit an invasive growth pattern along white matter architecture and vasculature. This study quantified lesion development patterns in treated glioblastoma lesions and their relation to white matter microstructure to distinguish TP from TRA.

**Materials and methods:**

Glioblastoma patients with confirmed TP or TRA with T1-weighted contrast-enhanced and DTI MR scans from two posttreatment follow-up timepoints were reviewed. The contrast-enhancing regions were segmented, and the regions were coregistered to the DTI data. Lesion increase vectors were categorized into two groups: parallel (0–20 degrees) and perpendicular (70–90 degrees) to white matter. FA-values were also extracted. To test for a statistically significant difference between the TP and TRA groups, a Mann‒Whitney U test was performed.

**Results:**

Of 73 glioblastoma patients, fifteen were diagnosed with TRA, whereas 58 patients suffered TP. TP had a 25.8% (95% CI 24.1%-27.6%) increase in parallel lesions, and TRA had a 25.4% (95% CI 20.9%-29.9%) increase in parallel lesions. The perpendicular increase was 14.7% for TP (95% CI 13.0%-16.4%) and 18.0% (95% CI 13.5%-22.5%) for TRA. These results were not significantly different (*p* = 0.978). FA value for TP showed to be 0.248 (SD = 0.054) and for TRA it was 0.231 (SD = 0.075), showing no statistically significant difference (*p* = 0.121).

**Conclusions:**

Based on our results, quantifying posttreatment contrast-enhancing lesion development directionality with DTI in glioblastoma patients does not appear to effectively distinguish between TP and TRA.

## Background

Glioblastoma is the most common malignant primary adult diffuse glioma. It grows invasively along white matter architecture and (micro)vasculature, known as perineural and perivascular satellitosis [1]. On MRI, glioblastoma presents as an intracerebral heterogeneous mass with central necrosis, an irregular contrast-enhancing rim and surrounding T_2_ hyperintense areas reflecting vasogenic edema and tumor infiltration. Due to its highly infiltrative nature, complete resection is practically impossible. The infiltration of satellite cells invariably leads to tumor growth and further disruption of the blood‒brain barrier, which in turn leads to the growth of contrast-enhancing regions on follow-up MRI, known as tumor progression (TP). This infiltrative growth pattern explains the high occurrence of incomplete resection and low overall survival of glioblastomas [2]. However, neurosurgery and concurrent chemoradiotherapy following the Stupp protocol can also lead to increased volumes of contrast-enhancing regions. These regions are identical in presentation to TP lesions on conventional MRI. Treatment-related abnormalities (TRA) include pseudoprogression and radiation necrosis and either remain stable over time or slowly resolve spontaneously. Differentiation of TP from TRA has thus far been challenging using conventional MRI, as these entities appear almost identical [3]. Nevertheless, differentiating TP from TRA is of paramount importance because patients who exhibit TRA have significantly better progression-free survival, Karnofsky performance score and overall survival [4, 5], and incorrect diagnosis results in early termination of effective treatments, unnecessary interventions or continuation of ineffective treatments [6].

Therefore, new diagnostic imaging tools that accurately distinguish TRA from TP are warranted. New imaging methods could focus on imaging the different biological properties of TRA and TP lesions, including lesion perfusion, the presence of oncometabolites and the transport of amino acids [7, 8]. Another biological property that has been hypothesized to differ between TRA and TP lesions concerns their respective patterns of development. Following the growth pattern of preoperative glioblastoma, TP lesions are hypothesized to elicit a perineural tumor growth pattern, corroborating histopathology findings [9]. TRA lesions, on the other hand, are hypothesized to show a more random pattern of volume increase of contrast-enhancing areas over time. Diffusion tensor imaging (DTI) could provide a tool for illustrating these developmental patterns, as it has the ability to probe preferential diffusion directions of water in white matter microstructures in the brain. Previous efforts to map *pre*treatment glioblastoma growth showed that DTI was capable of measuring the growth of the contrast-enhancing part of glioblastomas along white matter structures [10, 11]. Therefore, this study quantified the patterns of developing contrast-enhancing lesions in posttreatment glioblastoma patients in relation to white matter microstructure in an attempt to accurately distinguish TP from TRA.

## Materials and methods

### Ethical approval

The local ethical review board (METC Oost-Nederland) waived ethical approval due to the retrospective nature of this study (ethical review board assigned file number 2020–6480). Patient data were generously supplied by co-authors. Patients who were unwilling to share their data for research purposes were not included in this study, following institutional standard operating procedures with regard to retrospective studies. All patient data were anonymous and safeguarded according to secure data management principles.

### Study population

In this retrospective study, patients diagnosed with glioblastoma based on histopathological assessments and selected from December 2013 to August 2020 at the university clinic were considered eligible for inclusion. (*N* = 138) [12]. Patients were selected for this study if they had undergone two posttreatment MRI examinations during the follow-up period. Disease outcome was determined according to the reference standard, which was histopathology of biopsies. Both MRI investigations were performed on either Philips Ingenia 1.5 or 3T scanners (Philips Healthcare, Best, Netherlands), and a postcontrast T_1_-weighted imaging series and a DTI sequence were used; the details of the scanning protocol can be found in Table 1. Patients were excluded if (1) they were under 18 years of age, (2) they did not allow their data to be used for research, (3) they had a history of other neurological diseases, such as extensive infarction, meningioma or previous neurosurgical treatment, (4) their MRI scans were unavailable or not processable, (5) their DTI scan at the first timepoint was unavailable or (6) the quality of their MRI scans was suboptimal due to major differences in image quality or spatial resolution between the two timepoints, which could affect the accuracy of the deformation field estimation. DTI scans at the first timepoint were used to map white matter microstructure and to determine whether the lesion would show a perineural or a random pattern of development.

**Table 1:** Acquisition parameters

Sequence	3T T_1_w + C	3T DTI	1.5T T_1_w + C	1.5T DTI
Type	3D FFE	Spin echo EPI	3D FFE	Spin echo EPI
TR (ms)	7.5	3945	7.7	2655
TE (ms)	3.5	86	3.6	81
FA (deg)	8		8	
B-values	n/a	B = 0 B = 1000, 6 directions	n/a	B = 0 B = 1000, 6 directions
FOV (mm)	240 × 240 × 187	230 × 230 × 169	256 × 238 × 160	230 × 230 × 153
Spatial resolution (mm)	1.1 × 1.1 × 1.1	2.1 × 2.6 × 4	1.1 × 1.1 × 1.1	2.1 × 2.6 × 6
Acquisition time (min: s)	03:49	01:23	04:04	00:40

*Abbreviations* T1w + C: Contrast-enhanced T1-weighted, FFE: Fast field echo, DTI: Diffusion tensor imaging, EPI: Echo planar imaging, TR: Repetition time, TE: Echo time, FA: Flip angle, FOV: Field of view

### Preprocessing and image registration

All DICOM files were converted to NifTI files to accommodate processing in the Functional MRI of the Brain (FMRIB) Software Library (FSL). The DTI data were corrected for eddy currents using the FSL eddy correction tool [13], followed by skull stripping and the creation of a brainmask using the FSL bet2 tool [14]. Finally, the tensor images were calculated using FSL’s dtifit tool [15], which uses least-square fitting to determine the tensor, producing fractional anisotropy maps, mean diffusivity maps, eigenvectors and eigenvalues. The T_1_-weighted contrast-enhanced scan from the second timepoint was coregistered to the data from the first timepoint using a 12-point linear affine registration tool (FMRIB’s Linear Image Registration Tool, v6.0) [16]. The patient-specific DTI series from the first timepoint was then also normalized and coregistered to the T_1_-weighted images of the first timepoint to quantify the directionality of the increase in lesions in relation to the local white matter microstructure. An in-house written script (MATLAB R2022b v9.13.0) was used to extract Dxx, Dyy and Dzz tensor elements from the DTI series [17].

### Image segmentation and deformation field calculation

The changing contrast-enhancing lesions on images at the first and second timepoints were segmented using a combination of manual and semiautomatic methods via ITK-snap software, which is specifically designed for 3D medical image segmentation via active contour methods, manual delineation, and image navigation [18]. The primary investigator (R.E. with less than five years of experience with experimental neuroimaging) performed the preliminary segmentation. To ensure the accuracy of the segmentation procedures, a supervisor oversaw the segmentation process (D.H.; resident radiologist with more than eight years of experience with experimental and clinical neuroimaging). If there was a lack of agreement or uncertainty regarding the precision of the segmentation, the opinion of a board-certified neuroradiologist with 20 years of experience (F.M.) was sought.

**Fig. 1:**
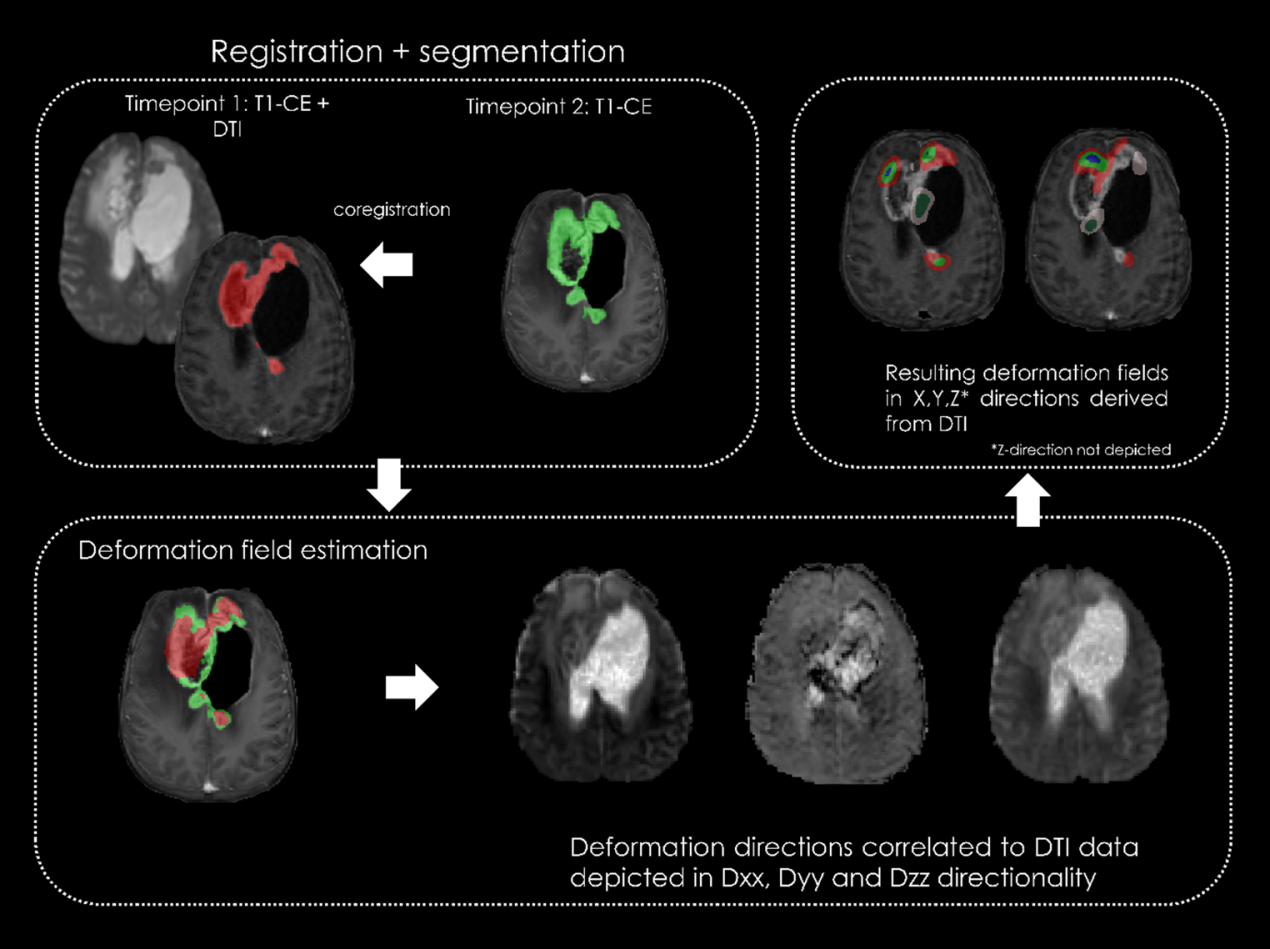
A simplified overview of the image registration and deformation method, coregistration of timepoint 2 contrast-enhanced data to timepoint 1 contrast-enhanced and DTI datasets. The segmentations are used for a deformation field estimation and overlaid on DTI tensors Dxx, Dyy and Dzz, with calculated deformation fields as the output. CE, contrast-enhanced; DTI, diffusion tensor imaging

Segmentations of the contrast-enhancing regions from both timepoints were made binary. Then, the first timepoint mask images were overlaid and registered to the second timepoint using the nonlinear function of the Advanced Neuroimaging Tool (ANT) script packages [19, 20]. The result was a deformation field characterizing directional changes in lesion shape and volume between the two timepoints. Using the ITK-SNAP tool Convert3D [18], this localized, multicomponent deformation field was converted into three orthogonal vectors. Using an in-house written MATLAB script, which was partially derived from previous growth deformation estimation studies [10, 17], the combination of DTI vectors and the obtained deformation field allowed for calculation of the voxelwise alignment between white matter structure directionality (represented by DTI vectors) and directional changes in lesion size (deformation field). This alignment was separated into bins of 10 degrees, ranging from 0 to 90 degrees in magnitude, where 0 degrees represents a parallel increase along white matter structure and 90 degrees represents a perpendicular increase. The amount of growth in each of the 9 bins was calculated as a percentage of the total increase per lesion. Following thresholds reported in previous studies [10, 17], vector bins of 0–20 degrees were defined as parallel increases, while 70–90 degrees were chosen to indicate perpendicular lesion increases with respect to white matter microstructure. To confirm or refute the hypothesis that TP lesions exhibit more aligned growth patterns with white matter than TRA lesions, which are anticipated to display a more random pattern of lesion increase, resulting in a greater percentage of perpendicular growth, this study will analyze the spatial distribution and orientation of lesions in both groups. A simplified overview of the image registration and deformation pipeline can be found in Fig. 1.

### Statistical analyses

Statistical analyses were performed using IBM SPSS software (version 27; IBM Corp., Armonk, NY). The normality of the distributions of vector groups between the TP and TRA groups was tested using an independent sample Kolmogorov‒Smirnov test. To test for statistical significance between the means of growth vector populations in the TP and TRA groups, a Mann‒Whitney U test was performed. To test for statistical significance between the parallel and perpendicular vector groups, a one-sample Student’s t test was performed. P values ≤ 0.05 indicated statistical significance. The fractional anisotropy (FA) value was also derived from segmented regions of interest (ROI) and compared to values reported in literature. Statistically significant difference of FA-value between TP and TRA groups was tested using a Mann-Whitney U test.

## Results

### Population

A consecutive cohort of 138 patients was screened for eligibility for inclusion in this study. Seventy-three patients met the inclusion criteria. Sixty-five patients were excluded from the original cohort due to unavailability of MRI scans (*N* = 26), missing DTI scans at the first timepoint (*N* = 34) or suboptimal quality MRI data (*N* = 5). Fifteen patients were histopathologically confirmed to have TRA, while fifty-eight patients were confirmed to suffer from TP. Average time between scans was 77 days with a 38-day SD.

### Vector population

The normality test showed that the mean lesion increase direction in the parallel and perpendicular groups was distributed normally between all patients, with a p value of 1.000 for the parallel (0° – 20°) vector group and a p-value of 0.158 for the perpendicular (70° – 90°) vector group.

Analysis of the complete patient population, including both TP and TRA data, revealed a mean percentage of 25.7% (95% CI 24.0% – 27.4%) of voxels with lesions increasing parallel to the white matter microstructure (0° – 20°). An increase in the percentage of perpendicular lesions in relation to white matter microstructure (70° – 90°) was observed in 15.4% (95% CI 13.8% – 17.1%) of the voxels. When comparing parallel to perpendicular increase patterns, there was a statistically significant difference between the two (*p* < 0.001, Cohen’s d 0.79), corroborating previous literature showing lesion growth in pretreatment glioblastoma and its preference for spreading along white matter [10, 17].

### Differentiation of TP vs. TRA

**Fig. 2:**
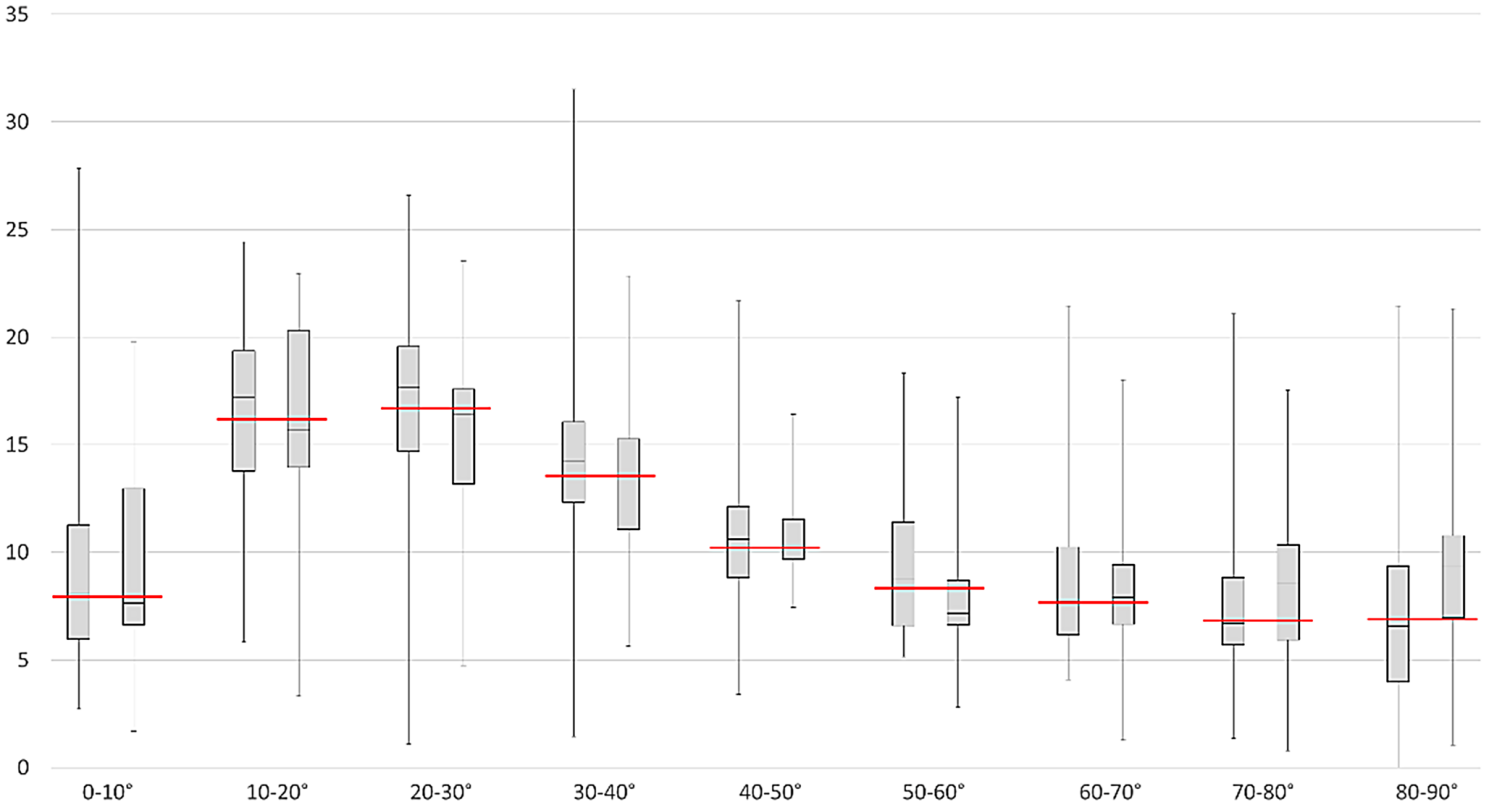
Left boxes: TP; right boxes: TRA; red line: median. There was large overlap observed among all vector populations, with the median values consistently aligning closely with the means of both groups. There was a greater degree of lesion increase between 10° and 40° in both groups, indicating a preference for developing into the preexisting space between microstructural white matter fibers

To assess whether lesion increase could distinguish between the TP and TRA, the mean parallel and perpendicular lesion increases in the TP group were compared to those in the TRA group. For TP, the mean parallel lesion increase was 25.8% (95% CI 24.1%-27.6%), and for TRA, it was 25.4% (95% CI 20.9%-29.9%). The mean perpendicular lesion increase was 14.7% for TP (95% CI 13.0%-16.4%), and for TRA, it was 18.0% (95% CI 13.5%-22.5%). The Mann‒Whitney U test showed insufficient evidence to confirm that the distributions of vector populations between the TP and TRA groups were different in parallel (*p* = 0.978) or perpendicular (*p* = 0.088) vector bins. Figure 2 shows the overlap between TP and TRA when using this method, as well as a more abundant increase in line with the white matter microstructure, especially between 10° and 40°, than between the higher vector groups. Mean FA value for TP showed to be 0.248 (SD = 0.054) and for TRA it was 0.231 (SD = 0.075). Mann-Whitney U test showed insufficient evidence for a significant difference in FA-value between TP and TRA (*p* = 0.121). This result differs from results in a recent meta-analysis, with a mean FA for TP of 0.19 and for TRA being 0.14, stating that FA-values could, on a group level, differentiate between TP and TRA [21].

## Discussion

The results of this study showed that both TP and TRA lesions tended to increase in volume in parallel to the white matter microstructure. Quantification of patterns of increasing contrast-enhancing lesions was unable to distinguish between TP and TRA. Together, these results suggest that contrast leakage in TP and TRA occurs in the same anatomical directions between the densely packed white matter bundles. This “*perineural compartment*” provides a constitutive trail for glioma cell migration and allows fluid circulation [22, 23]. Regarding glioma cell migration, the extracellular matrix of the brain contains large quantities of specific molecules (e.g., neurocan and brevican (lectins) and phosphocan), which are secreted by astrocytes and oligodendrocytes and play a promigratory role [24, 25]. Additionally, glioma cells produce proteins (e.g., brevican and tenascins), which increase their invasiveness [26–28]. With regard to the leakage of fluids in the perineural space in TRA, given the white matter microstructure, this leakage has already been described by Joseph Klingler, an Austrian anatomist, in the 1930s [29]. Klingler described that a lipophobic 5% aqueous formaldehyde penetrates the brain parenchyma, though it remains in the perineural compartment. It is also noted that TRA is likely triggered by a significant local tissue response, where inflammation, edema and abnormal vessel permeability lead to the emergence or intensification of contrast enhancement on neuroimaging [30], which, in turn, is able to diffuse along the white matter microstructure. This could explain why TRA lesions also develop parallel to the white matter microstructure, as contrary to what we hypothesized, in TRA contrast agent appears to leak into the perineural space and is not affected as randomly by the irradiation field alone. It should be noted that in the clinical setting, contrast fluid diffuses in approximately 10 min, giving the radiologist a limited view of the total diffusion capacity of the lesion, as directional diffusion may take longer than the time needed to acquire an MR scan. Current results corroborate previous findings [10, 17], where contrast-enhancing parts of glioblastoma in the pretreatment setting seem to follow white matter orientation and grow more parallel rather than perpendicular. This alignment with the white matter microstructure offers insight into the intricate dynamics of glioblastoma growth in accordance with previous literature [9], as well as insight into lesion changes in TRA. These results of lesion growth may aid in future modeling of tumor growth in the pretreatment setting, providing information on lesion expansion in the brain.

Regarding the FA-value, our results showing no distinct difference between TP and TRA groups contradict earlier results presented in a meta-analysis. The mean difference in our cohort lies below 0.02, with insufficient evidence from the Mann-Whitney U test to state there is a significant difference between groups. This corroborates the conclusion that on an individual level, FA is not able to distinguish between TP and TRA, due to the lack of validated diagnostic criteria on an individual level and harmonization of data [21, 31].

One notable strength of this study is the use of patient-specific data. Unlike previous studies that relied on template-based diffusion tensor imaging data, this research incorporated individual patient data for white matter microstructure definition. This approach provides a more accurate representation of the unique characteristics of glioblastoma in each patient as opposed to using a DTI atlas. However, this methodology produces roughly the same results as previous studies with the DTI atlas. The large slice thickness (6 mm) of current patient-specific DTI should be taken into account as it sets up for a margin of error, being less sensitive to smaller and more tortuous tracts, though it is not known what the eventual effect of anisotropy is on our outcome measure. Moreover, besides the lack of isotropic resolution in DTI, the lack of more advanced complementary neuroimaging techniques such as diffusion kurtosis imaging (DKI) represents a missed opportunity to obtain a more comprehensive characterization of tissue microstructure between TP and TRA. Though they are not (yet) part of clinical routine, sequences using DKI extend DTI by also measuring non-Gaussian diffusion, representing deviations from a Gaussian distribution of water diffusion. The quantified degree of diffusion kurtosis signifies tissue complexity due to being more sensitive to microstructural heterogeneity such as areas of crossing fibers or tumorous regions [32, 33]. Additionally, the low number of diffusion directions (*N* = 6) could also affect our results, however the potential bias is reduced by categorizing voxel directions in bins of 10°. Other advanced metrics and sequences such as high angular resolution diffusion imaging (HARDI) with more diffusion directions to better evaluate fiber tract direction or q-ball imaging to assess regions of complex fiber orientations could provide clinicians with other information depending on the region of interest [34, 35]. However, a higher number of diffusion directions or b-values result in significantly longer measurement times, which are often not feasible in clinical practice. Using a validated pipeline from previous studies [10, 17] for image processing is another strength of this study and enhances replicability. By using an established and validated methodology, the study ensures the reliability and reproducibility of its results. Additionally, the histopathological validation of all the contrast-enhancing lesions is a strength of this paper. The small number of patients diagnosed with TRA lesions might have affected the results of the growth direction analysis due to inaccuracy with a limited sample size. The fact that only a selection of ROIs was reviewed by a board certified neuroradiologist might have led to an underestimation of effects as an experienced reader may select which area of enhancement would be most informative for the analysis. However, this impact is considered minimal as the here applied method called for the entire contrast-enhancing region to be selected. Another limitation of this study was that other growth patterns of glioblastoma lesions, including subependymal spreading, seeding and/or perivascular invasion, could not be taken into account. Future studies with larger cohorts could help address this limitation. An important limitation inherent to DTI to take into account is the reliability of the white matter microstructure definition due to tumorous changes, blood, and edema. Over time, the volume of surgical cavities and surrounding edema typically tend to change in size [36, 37]. However, the deformation pipeline and DTI data fail to account for these reductions in lesion volume, limiting their practicality. Another related potential drawback this quantification method could not account for is how well the quantification of parallel and perpendicular voxels represent the growth of potential tumor in relationship with the white matter tracts. On one side, the deformation of contrast enhancement mask may represent both the mass effect and lesion infiltration. In the case of mass effect, the percentage of parallel lesion growth may depend heavily on the location of the lesion in the brain, rather than the hypothesized perineural growth pattern. On the other side, the directionality of white matter fiber might be less reliable in gray matter regions and non-enhancing infiltrative tumor regions, which may affect the accuracy of the computed alignment angles. Literature has proposed sophisticated tractography techniques, such as probabilistic tractography, as a possible method for forecasting the spread of glioblastoma. Nonetheless, it’s crucial to acknowledge that tractography might generate white matter tracts that appear feasible, yet may not correspond to actual anatomical structures., however, it is important to note that tractography may produce white matter tracts which look possible, but in reality do not actually exist [38, 39]. Another potential limitation concerns the interobserver variability in determining the ROI for contrast-enhancing tumors on MRI, although two researchers (RE and DH) oversaw the segmentation process and a board-certified neuroradiologist (FM) was consulted in the case of disagreement between researchers. While this study provides insights into the development of TRA contrast-enhancing lesions in glioblastoma, it is important to note that the generalizability of these findings to other glioma subtypes, such as low-grade gliomas or other high-grade gliomas, remains uncertain. The unique molecular and histopathological characteristics of different glioma types may lead to variations in tumor growth patterns, warranting further investigation to determine the applicability of these observations in lower grade gliomas.

## Conclusion

This study showed that quantifying lesion growth directionality is not a reliable method for distinguishing between TP and TRA in posttreatment glioma patients, as both types of lesions tend to prefer to extend parallel to the white matter microstructure.

## Data Availability

The datasets used and/or analysed during the current study are available from the corresponding author upon reasonable request.

## Abbreviations

DTI: Diffusion tensor imaging
DICOM: Digital Imaging and Communications in Medicine
FLIRT: FMRIB’s Linear Image Registration Tool
FMRIB: Functional MRI of the Brain
FSL: FMRIB’s Software Library
IDH: Isocitrate dehydrogenase
ROI: Region of interest
TP: Tumor progression
TRA: Treatment-related abnormalities
WHO: World Health Organization
